# A Rare Case of Omental Strangulation Through the Esophageal Hiatus Successfully Repaired Through a Robotic-Assisted Approach

**DOI:** 10.7759/cureus.79245

**Published:** 2025-02-18

**Authors:** Gerard V Giangrosso, Juan G Bastidas

**Affiliations:** 1 General Surgery, Marshall University Joan C. Edwards School of Medicine, Huntington, USA

**Keywords:** computed tomography, magnetic resonance image, omentum, robotic-assisted surgery, robotic hiatal hernia

## Abstract

A 65-year-old male presented to the emergency department complaining of 48 hours of severe epigastric pain and guarding on physical exam. A non-contrast CT revealed a retrocardiac lipomatous mass. Chest MRI revealed an intrathoracic omental hernia through the esophageal hiatus. Intraoperatively, strangulated omentum was seen; specifically, the greater omentum herniated through the defect into the esophageal hiatus. The hiatal hernia was then successfully repaired via a robotically assisted, laparoscopic approach, and an omentectomy was performed.

## Introduction

A hiatal hernia occurs when part of the stomach, intestines, or omentum protrudes into the thoracic cavity through the esophageal hiatus of the diaphragm. Embryologic development of the diaphragm is a complex process; several errors could result in a variety of possible congenital hernias through the diaphragm. However, acquired hernias through the esophageal hiatus are more common. Most hiatal hernias are found incidentally, usually discovered on routine chest radiographs or computed tomography (CT) scans performed for unrelated symptoms. Rarely, a sliding hiatal hernia may present acutely because of a volvulus or strangulation [1].

Hiatal hernias are commonly seen among the general population. In one study performed by Loffeld et al, 19.9% of patients that underwent an esophagogastroduodenoscopy (EGD) were found to have a hiatal hernia [2]. Another study found the prevalence of hiatal hernias to be anywhere between 10-50% amongst the studied population [3]. Classically, the organs that herniate through the diaphragmatic hiatus include the stomach, colon, and spleen. Omental herniation through the esophageal hiatus is rare, mostly due to the difficulty of the omentum to slip superiorly above the stomach and colon. Those cases do occur are mostly asymptomatic [4]. Despite this, we discovered a case in which the patient was very symptomatic due to the strangulation of the omentum.

Early diagnosis of an omental hiatal hernia is difficult since most are asymptomatic; however, when symptomatic, abdominal pain is the main symptom. Contrast-enhanced abdominal computed tomography (CT) scan is recommended for early revelation of direct and indirect signs [5]. However, when there are questionable findings on the CT scan, an MRI can be used assist with diagnosis.

 Like other type IV hiatal hernias, these omental hiatal hernias could historically be fixed either open or laparoscopically. However, with the advent of the Da Vinci Xi Robotic System (Intuitive Surgical, Inc. Sunnyvale, CA), patients are now getting the option of a robotic repair for many thoracic and foregut surgical procedures. We report a rare case of a strangulated omental hiatal hernia that required an MRI of the abdomen to confirm the diagnosis. We also report our findings to encourage robotic-assisted, laparoscopic repair as a suitable option for the repair of this type of hernia due to improved precision and visualization.

## Case presentation

**Figure 1:**
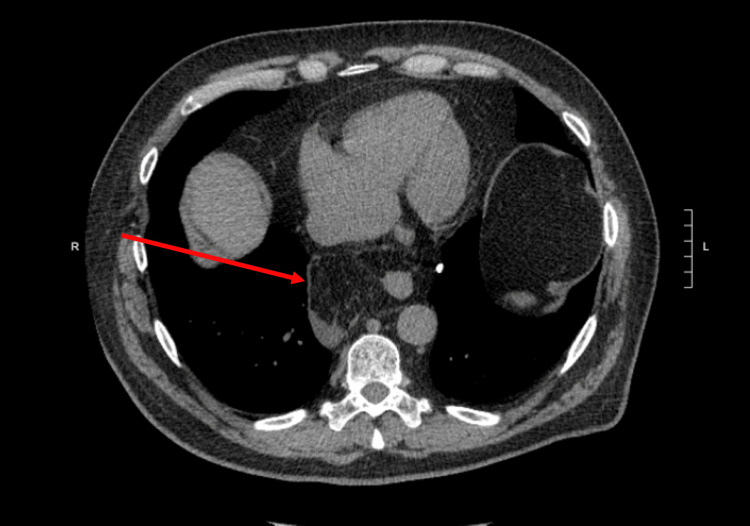
Transverse view, lipomatous retrocardiac mass with some fluid collection around the mass consistent with strangulation

**Figure 2:**
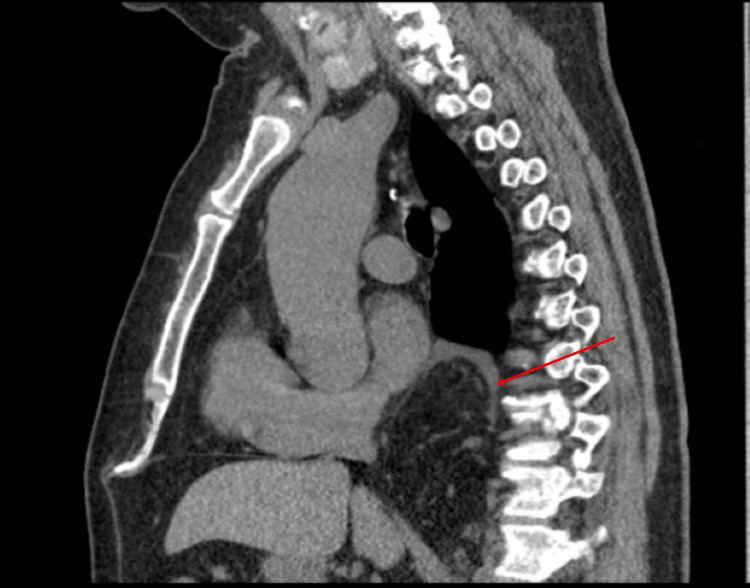
Sagittal view of the retrocardiac mass

**Figure 3:**
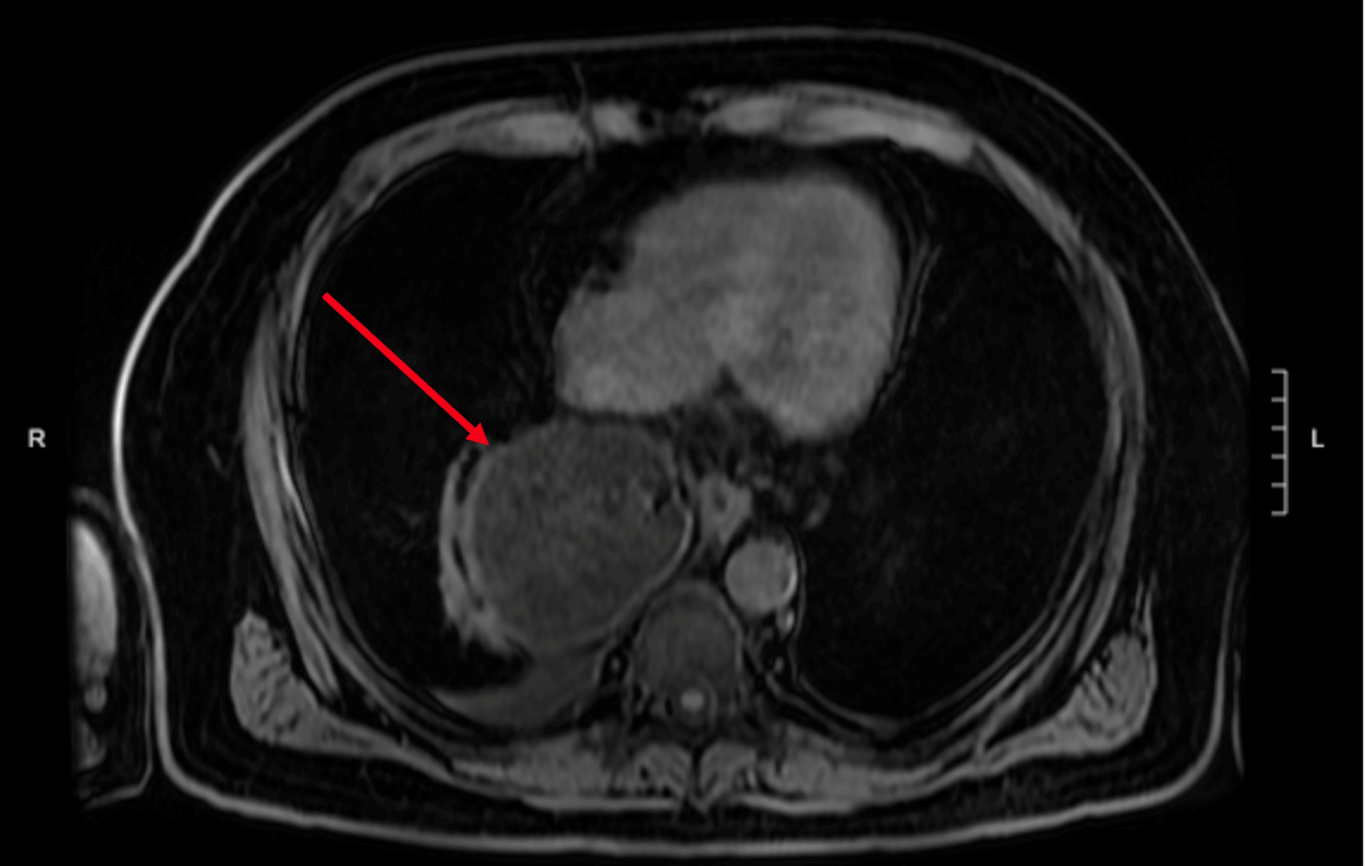
Transverse view of the intrathoracic omentum

**Figure 4:**
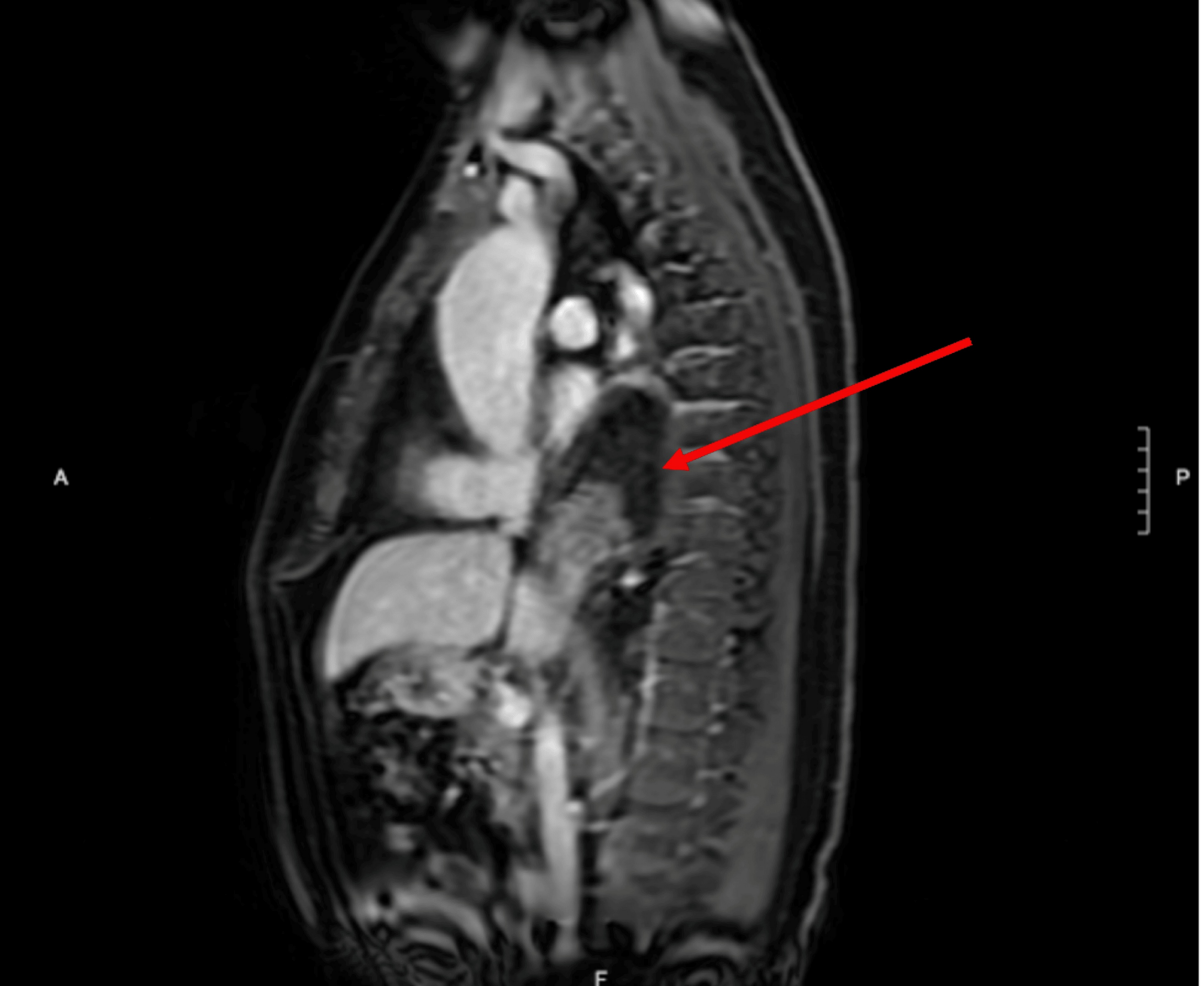
Sagittal view: the herniation is seen in the dorsal side of the diaphragm

A 65-year-old male presented to the emergency department with a chief complaint of 48 hours of epigastric pain radiating to the back. Past medical history was significant for a 5 cm ascending aortic aneurysm. The patient had no past surgical history or significant family history. He was afebrile, normotensive, and tachycardic. On physical exam, the upper abdomen was tender to palpation with guarding but no rigidity or rebound tenderness. A non-contrast helical abdominal CT scan revealed a soft tissue mass just above the gastroesophageal junction with the differential diagnosis of a lipoma or liposarcoma (Figures 1-2). Since the diagnosis was still not clear, an MRI of the chest was ordered. It revealed an intrathoracic omental hernia through the esophageal Hiatus (Figures 3-4) and fluid accumulation around the herniated omentum suggesting strangulation. In the sagittal view of the MRI, one can appreciate the omentum from the abdomen getting into the thoracic cavity (Figures 2-4). 

**Figure 5:**
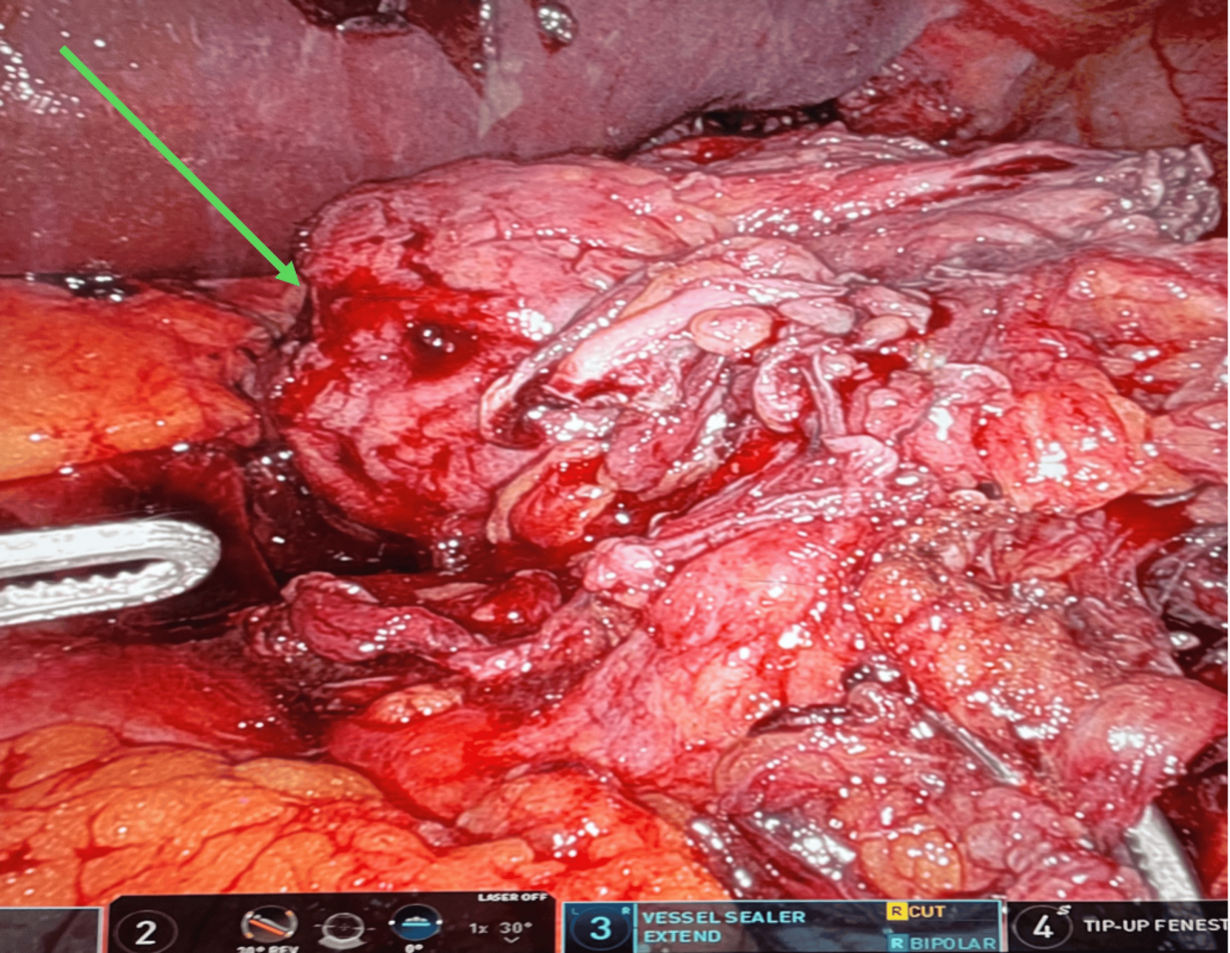
Strangulated omentum reduced from intrathoracic position

**Figure 6:**
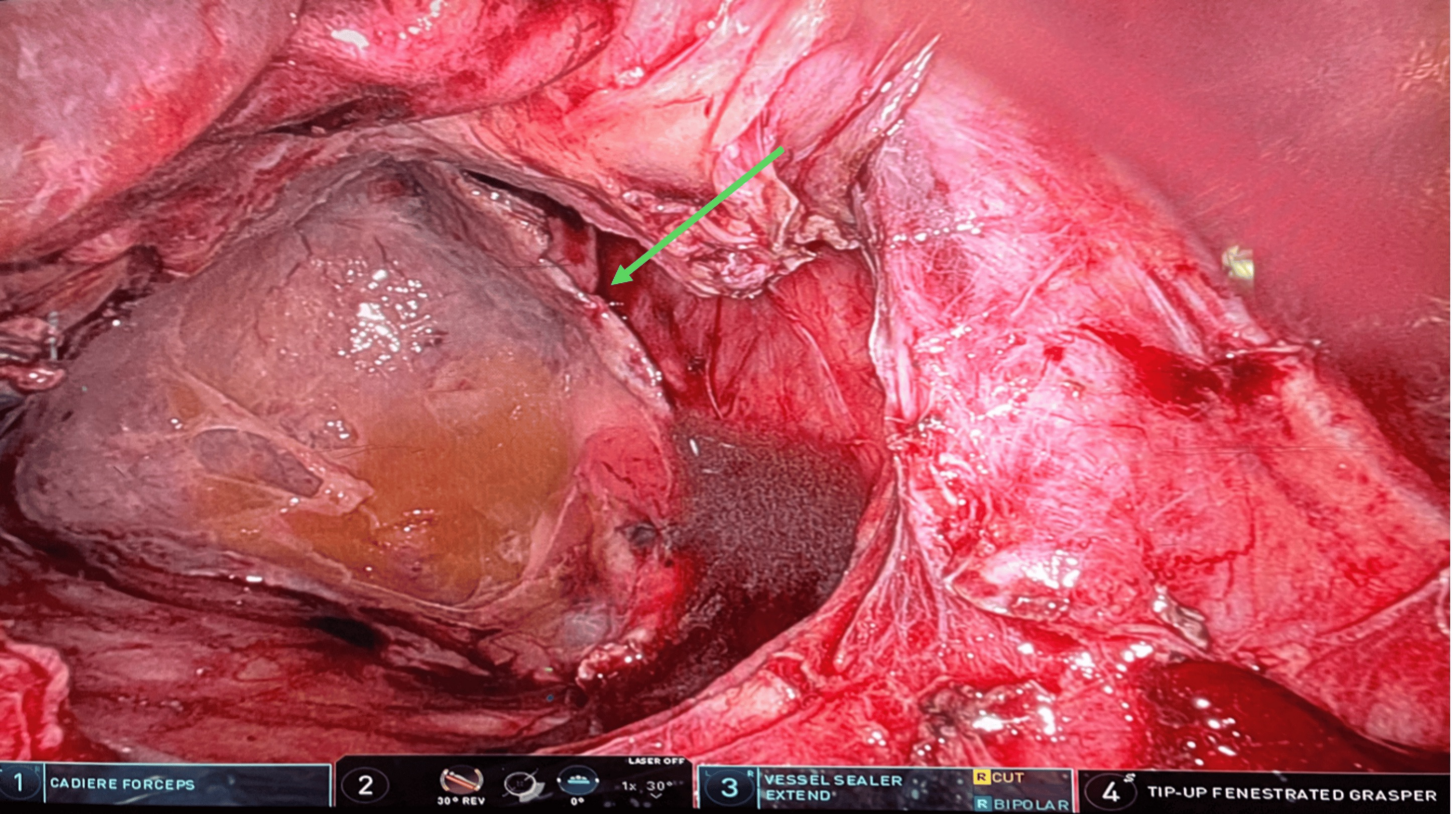
Hiatal defect larger than 8 cm

At this point, the decision was made to bring the patient to the operating room using the Da Vinci Xi Robotic System to carry out a robotic-assisted, laparoscopic approach. After induction of general anesthesia, the robotic cart was placed on the left side of the patient, and the ports were placed in the same fashion as an elective hiatal hernia repair. We placed the camera through the supra-umbilical port, the cadiere forceps through the left port, the vessel sealer through the middle-right port, and the tip-up fenestrated grasper through the right port. In this case, we also placed an accessory 5-mm sub-xiphoidal port to retract the liver, as common in all our robotic repairs. The vessel sealer was used to lyse the adhesions of the hernia sac from the mediastinal structures, while the herniated omentum was gently reduced in the abdomen. Once reduced, an omentectomy was performed of the strangulated omentum (Figure 5). The sac was excised, and the diaphragmatic crura and esophagus were isolated. The hiatal defect was approximately 8 cm in diameter (Figure 6). The esophageal hiatus was then repaired with a biological mesh BIO-A (Gore & Associates Inc., Newark, DE, USA). Biologic mesh was used due to the presence of necrotic omentum and concern for possible infection. The patient had an uneventful recovery, and he was discharged on postoperative day 3 after tolerating the diet without any complications.

## Discussion

Strangulated omental herniation through the esophageal hiatus is rare. The elasticity of fibers of the diaphragmatic crura decreases in people over 50 years of age increasing the risk of herniation, but this case is still a very uncommon occurrence [6]. Historically, lipomatous tumors have been misdiagnosed as omental hernias, but when there is diagnostic uncertainty, an MRI is a very helpful diagnostic tool. In this case presentation, the MRI of the chest gave a diagnosis of omental hiatal hernia [7].

There are several cases of incarcerated omentum through the diaphragmatic hiatus that were initially misdiagnosed as a mediastinal lipoma or other mediastinal mass. Upon operative intervention, they were later found to be incarcerated omentum. Kato et al. described a 10 x 7.5 x 6 cm omental herniation through the esophageal hiatus, which did not involve any stomach or any parts of the small or large bowel. The omentum was resected, the hiatus was approximated, and the patient was discharged with no complications [8]. Maruyama et al described a similar presentation of a 21-year-old male who presented with a 17 x 12 x 8 cm mediastinal mass, later found to be omental herniation, who underwent resection and repair. Again, there was no stomach or colon involvement [9,10]. All the cases underwent repair of the hernia defect and resection of the omentum with no complications. These cases emphasize the fact that incarcerated/strangulated omentum should be considered when dealing with any herniation through the diaphragmatic hiatus in addition to the other organs like the colon, spleen, and pancreas. Moreover, any mediastinal mass discovered on imaging near the gastroesophageal junction should include incarcerated/strangulated omentum in the differential diagnosis.

A case like this could have been done using conventional laparoscopy or even as an open procedure. Historically, any concern for strangulation would necessitate an open procedure, with only the laparoscopic experts considering fixation with a laparoscope. However, the Da Vinci Robotic System allows for a much easier time dissecting the omentum and repairing the hernia with precision. It also has the added benefit of giving patients a smaller incision with improved healing. Our case was made much simpler with the robot, and in the future, robotic repair should be considered when there is a concern for strangulated omentum within a hiatal hernia.

## Conclusions

Our patient initially presented with severe epigastric pain and was initially misdiagnosed with a retrocardiac lipomatous tumor. With the help of MRI, we were able to better visualize a fatty structure with contiguous blood vessels extending from the abdominal cavity into the thorax, and this led us to diagnose the patient with a strangulated paraesophageal omental hiatal hernia. Once this was realized, the patient was brought back for prompt surgical intervention with assistance from the Da Vinci Xi Robotic System. A robotic-assisted approach in an acute paraesophageal hernia repair conveys advantages in precision and visualization when compared to conventional laparoscopy or an open procedure. In the future, robotic repair should be considered when there is possible evidence of omentum involved with a hiatal hernia.
